# Molecular screening of *Amblyomma* species (Acari: Ixodidae) imported from African countries to Egypt, with the first report of *Amblyomma latum* from the ball python, *Python regius* (Squamata: Pythonidae)

**DOI:** 10.1007/s10493-023-00829-9

**Published:** 2023-08-08

**Authors:** Eman M. Abouelhassan, Marwa S. Kamel, Lidia Chitimia‑Dobler, Deon K. Bakkes, Mohammed Okely

**Affiliations:** 1 Department of Parasitology, Faculty of Veterinary Medicine, Suez Canal University, Ismailia, 41522 Egypt; 2 Department of Plant Protection, Faculty of Agriculture, Suez Canal University, Ismailia, 41522 Egypt; 3 Bundeswehr Institute of Microbiology, Neuherbergstrasse 11, Munich, Germany; 4 Gertrud Theiler Tick Museum - EPV, Onderstepoort Veterinary Research, Agricultural Research Council, Pretoria, South Africa; 5 Entomology Department, Faculty of Science, Ain Shams University, Abbassia, Cairo 11566 Egypt

**Keywords:** *Amblyomma* spp., Snake tick, Dromedary camels, Egypt

## Abstract

*Amblyomma* species are non-endemic ticks in Egypt, which have been recorded from imported animals. This study was carried out in 2022 to monitor *Amblyomma* spp. from dromedary camels, cattle, and snakes in Egypt. During this study, 400 camels, 200 cattle, and two snakes (Pythonidae) were inspected for tick infestation. Collected specimens were identified based on morphological characters and confirmed by phylogenetic analysis of the 12S rRNA gene. Camels were infested by adult specimens of *Amblyomma variegatum* and *Amblyomma lepidum*, but no *Amblyomma* spp. were collected from cattle. *Amblyomma variegatum* showed high genetic similarity to other *A. variegatum* from Guinea-Bissau and São Tomé (> 99.99%), and *A. lepidum* showed high genetic similarity to other *A. lepidum* from Israel and Sudan (99.99%). *Amblyomma latum* is recorded in Egypt from the ball python snake for the first time and showed high genetic similarity with South African *A. latum* (99.87%).

**Supplementary Information:**

The online version contains supplementary material available at 10.1007/s10493-023-00829-9.

## Introduction

The genus *Amblyomma* is represented by approximately 138 species worldwide, ca. 20% of all world tick species (Guglielmone et al. 2015; Okely et al. 2022). Species of this genus are distributed in Neotropical, Afrotropical, and Australasian regions, with the highest diversity in the Neotropical region (Barker and Burger 2018; Horak et al. 2018). A wide range of pathogens has been reported in ticks from this genus, such as *Borrelia* spp., *Ehrlichia* spp., *Rickettsia* spp., and *Anaplasma* spp. (Ogrzewalska et al. 2019; Jiang et al. 2021; Saijuntha et al. 2021; Qiu et al. 2021; Vieira et al. 2022).

In Egypt, *Amblyomma* ticks are represented by only six non-endemic species (Okely et al. 2022). In neighboring regions, *Amblyomma* spp. have been collected from domestic animals such as dromedary camels and cattle in countries such as Sudan, Ethiopia, Nigeria, and Somalia (Okely et al. 2021). These species are *Amblyomma eburneum*, *Amblyomma gemma*, *Amblyomma hebraeum*, *Amblyomma lepidum*, *Amblyomma marmoreum*, and *Amblyomma variegatum* (Robinson 1926; Hoogstraal 1952, 1956; El Kammah et al. 2001, 2007; Ghoneim et al. 2017, 2020; Elhelw et al. 2021; Okely et al. 2021). *Amblyomma eburneum* was reported in Egypt from imported cattle to Cairo only once (Robinson 1926). Ghoneim et al. (2017, 2020) recorded *A. hebraeum* from imported dromedary camels to Basatin abattoir, Cairo. *Amblyomma marmoreum* was recorded by El Kammah et al. (2001, 2007), but without data about its host. *Amblyomma gemma*, *A. lepidum*, and *A. variegatum* have been reported in Egypt in several studies (Hoogstraal 1952, 1956; Youssef et al. 2015; Ghoneim et al. 2017, 2020; Elhelw et al. 2021; Okely et al. 2021).

The snake tick, *Amblyomma latum*, is a hard tick species with an Afrotropical distribution, especially prevalent in sub-Saharan African countries (Guglielmone and Robbins 2018). Several families of squamates have been recorded as the principal hosts for all parasitic stages, with immature stages also reported from rodents (Muridae), and adults occasionally collected from anurans, tortoises, rodents, and soricomorphs (Guglielmone et al. 2014; Horak et al. 2018).

In the present study, we confirm the presence of *Amblyomma* species in Egypt using morphological and molecular identification and estimate phylogenetic relationships between the recorded species in Egypt and other *Amblyomma* spp. from GenBank records. Additionally, we report the first record of *A. latum* infesting a snake in Egypt.

## Materials and methods

In 2022, several collection trips were conducted to monitor *Amblyomma* tick species from domestic and wild animals in Egypt. In total 600 domestic animals (400 dromedary camels and 200 cattle), and two snakes belonging to family Pythonidae were inspected for *Amblyomma* species infestation. Camels, cattle, and snakes were examined monthly, twice, and only once, respectively. Camels were examined in the Birqash camel market, cattle were examined in cattle farms, and snakes were examined in markets for live snake trade (Figure S1). The collection sites of each animal were georeferenced using ArcGIS v.10.3 mapping software (Esri, Redlands, CA, USA) (Table 1).

**Table 1:** Detailed description of collection sites and hosts for monitoring *Amblyomma* species in Egypt

Host	Governorate	Longitude (N)	Latitude (E)
Dromedary camels	Giza	30.9953	30.1488
Cattle	Beni-Suef	31.15608	29.13925
Cattle	Sharkia	31.34741	30.51736
Snake	Faiyum	30.7866	29.2246
Snake	Matrouh	27.3799	29.4413

All *Amblyomma* spp. specimens were removed from animals using fine forceps and then stored in vials containing 70% ethyl alcohol for preservation. Drops of 20% glycerol were added for morphological identification in the Parasitology Laboratory, Department of Entomology, Ain Shams University. Specimens were identified to the species level based on morphological characters using diagnostic characters and identification keys (Hoogstraal 1956; Voltzit and Keirans 2003; Walker et al. 2003; Okely et al. 2021). Morphological characters were observed using a CZM4 Stereo Microscope (Labomed, Fremont, CA, USA) with an Am Scope LED-144 W-ZK white adjustable luminance. Species were photographed using an Am Scope MU1000 10MP Microscope camera (Am Scope, Irvine, CA, USA). Specimens preserved in 70% ethanol were processed for extraction of DNA. DNA was extracted utilizing QIAamp DNA Mini Kit (Qiagen, Hilden, Germany), following the manufacturer’s instructions. DNA was stored at -20 °C until use. PCR targeted amplification of the 12S rRNA gene using primers (5´-GAGGAATTTGCTCTGTAATGG-3´ and 5´-AAGAGTGACGGGCGATATGT-3´) according to Norris et al. (1999).

Amplified PCR products were excised from agarose gel, purified, and sent for sequencing. Sanger sequencing was performed by Solgent (Daejeon, South Korea). Sequences were then analyzed using BLAST (Johnson et al. 2008) to confirm they belonged to *Amblyomma*. Sequence data (GenBank acc. nrs. OP775457, OP785088, OP785089, and OP783984) were compiled with GenBank reference data to represent Afrotropical and related *Amblyomma* (32 sequences). Misidentified tick species in GenBank sequence data is a growing problem that can only be addressed with relatively complete taxon sampling of the underlying evolutionary tree. Phylogenetic tree estimation of the collected *Amblyomma* tick species from Egypt and other *Amblyomma* species available at the GenBank database used the hard tick *Ixodes* spp. (L43903, MF095802, and MF095798) as outgroup taxa. Sequence data were aligned using MAFFT (Q-INS-i, 200PAM/k = 2; gap opening penalty, 1.53) (Katoh et al. 2002). The optimal nucleotide substitution model was selected using BIC calculations in MEGA v.7.0.14 (Kumar et al. 2016) and was determined as TN93 + G with 5 gamma rate categories. Maximum likelihood analysis was performed in MEGA using 1000 bootstrap replications.

## Results

### Morphological identification

**Fig. 1:**
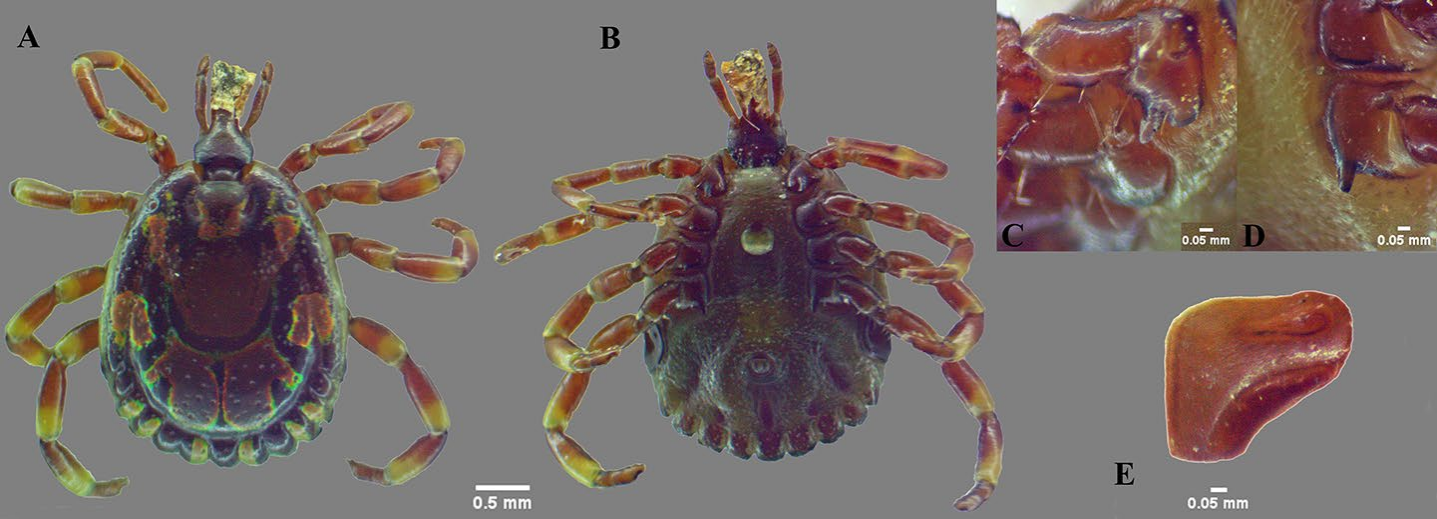
*Amblyomma lepidum* male: (**A**) dorsal view; (**B**) ventral view; (**C**, **D**) coxae; (**E**) spiracle

**Fig. 2:**
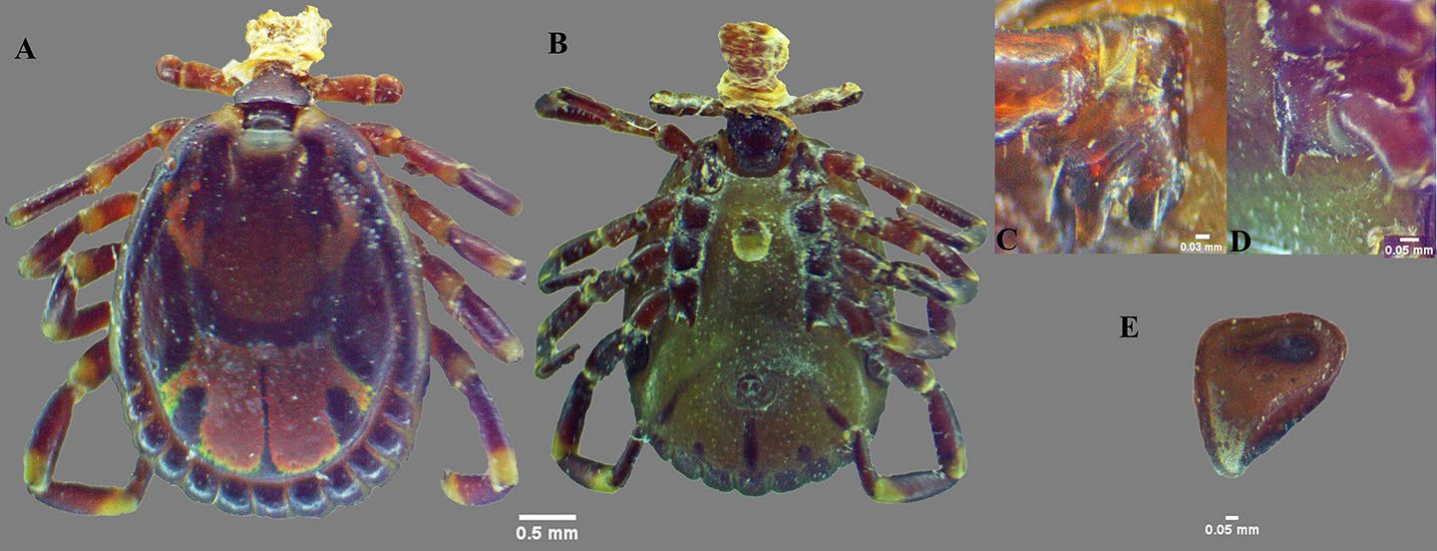
*Amblyomma variegatum* male: (**A**) dorsal view; (**B**) ventral view; (**C**, **D**) coxae; (**E**) spiracle

In total 66 individual *Amblyomma* ticks were collected (Table 2). Camels were infested by adult *A. lepidum* and *A. variegatum*. One of the two snakes was infested by only one *A. latum* nymph. The key diagnostic morphological characters that differentiate *A. lepidum* from *A. variegatum* are: mesial area of enamel ornamentation with dense, coarse punctations in *A. lepidum*, but smooth in *A. variegatum*; lateral median areas of enamel ornamentation large in *A. lepidum*, while absent in *A. variegatum*; festoons with enamel on 6–8 festoons of 11 festoons in *A. lepidum*, whereas the festoons are without enamel in *A. variegatum*; medial and posterior margins of the spiracular plate slightly straight, dorsal prolongation relatively broad, nearly perpendicular to axis of spiracular plate in *A. lepidum*, whereas medial and posterior margin of spiracular plate straight, dorsal prolongation triangular, very narrow, forming an obtuse angle with spiracular plate axis in *A. variegatum* (Figs. 1 and 2).

**Table 2:** *Amblyomma* tick species collected in Egypt during 2022

Host	Species	No. specimens	Life stage
Dromedary camels	*A. lepidum*	64	male
	*A. variegatum*	1	male
Cattle	*-*	-	-
Snakes	*A. latum*	1	nymph

### Phylogenetic analysis

**Fig. 3:**
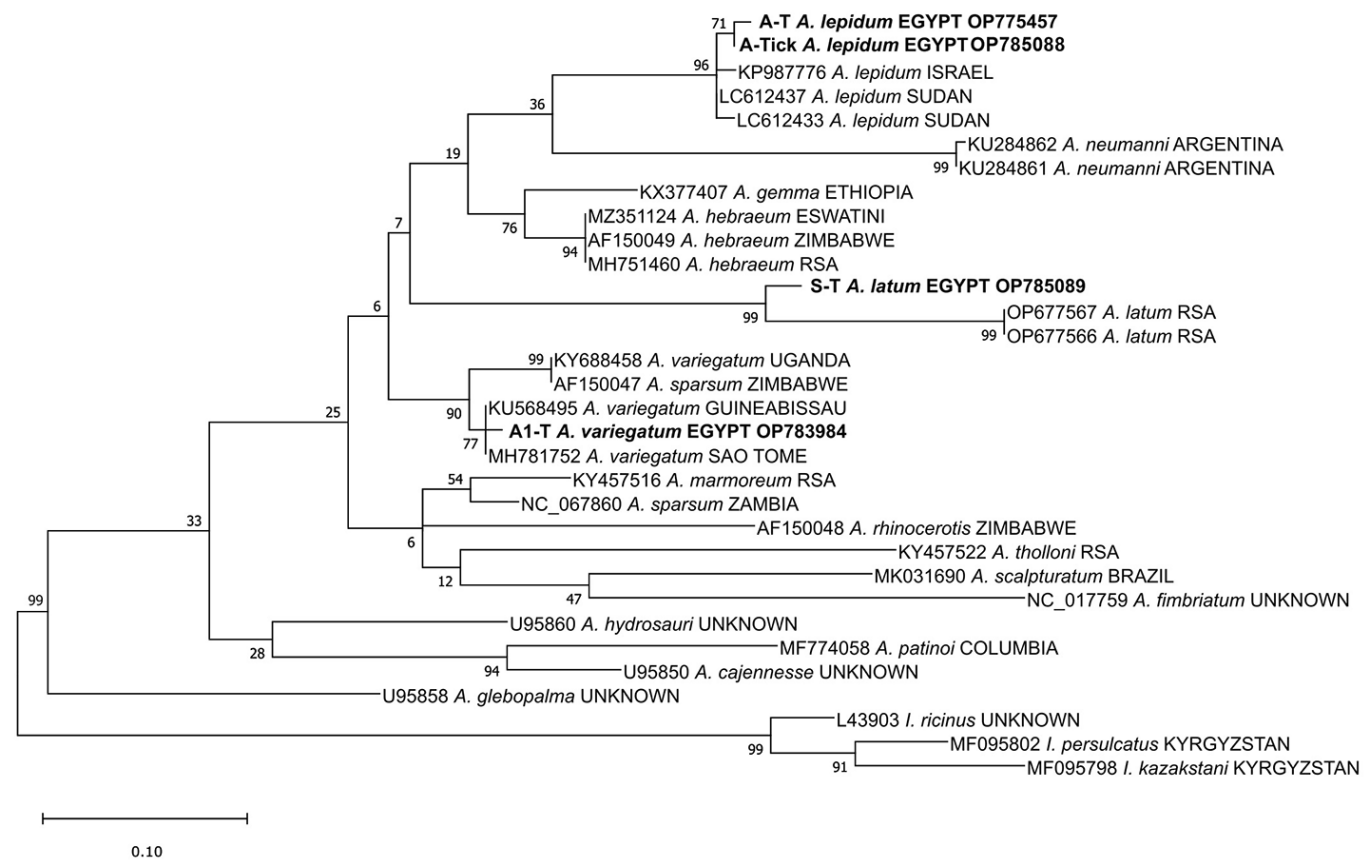
Phylogenetic relationships of *Amblyomma lepidum*, *A. variegatum*, and *A. latum* recorded from Egypt and other tick species based on 12S rRNA sequences

Tick species identifications were confirmed by phylogenetic analysis of the 12S rRNA gene. Lengths of amplified 12S rRNA sequences in all examined *Amblyomma* species in Egypt were found to be similar (approx. 300 bp); the length of the alignment was ca. 250 bp after trimming the low-quality ends of each sequence. The 12S rRNA sequences of *A. lepidum* collected from imported camels showed 99.99% similarity and were shown as a monophyletic group with specimens of *A. lepidum* from Israel (KPU987776) and Sudan (LC612437 and LC612433) (bootstrap support: 96%) and formed an independent, but weakly supported, sister clade with *A. neumanii* (KU284862 and KU284861) from Argentina with a bootstrap value of 36. Sample of *A. variegatum* from Giza governorate collected from imported camels showed > 99.99% similarity and formed a monophyletic group to other *A. variegatum* from Guinea-Bissau (KU568495) and São Tomé (MH781752) (bootstrap support: 77), in a clade sister to both *A. variegatum* (KY688458) and *A. sparsum* (AF150047) (indicating potential misidentified GenBank data). *Amblyomma latum* 12S rRNA sequences from Faiyum governorate and collected from imported ball python snake showed 99.87% similarity. They formed a monophyletic group to other *A. latum* (OP677567 and OP677566) reported from South Africa, albeit with some distinct phylogenetic distance (bootstrap support: 99) (Fig. 3).

## Discussion

Correct species identification of tick specimens is a crucial step to better understanding tick ecology, host-use patterns, life cycles, and epidemiology (Estrada-Peña et al. 2017; Okely et al. 2021; Perveen et al. 2021). Identifying ticks to species level may be based on morphological characters and molecular markers including 16S rRNA, 12S rRNA, cox1, 18S rRNA, 28S rRNA, ITS1 rDNA, and ITS2 rDNA (Cruickshank 2002; Nava et al. 2009; Araya-Anchetta et al. 2015; Estrada-Peña et al. 2017). In this study, we identified *Amblyomma* species recorded in Egypt using morphological characters and sequence data for the 12S rRNA gene. Although *A. latum* could not be identified using morphological features due to specimen damage, it was identified using molecular data in a phylogenetic context. As such, molecular tick identification helps overcome some difficulties associated with morphological identification (Abouelhassan et al. 2019).

In the present study, *A. lepidum* and *A. variegatum* infested imported camels; the highest number of specimens collected were *A. lepidum*. Our findings are in agreement with previous studies (Abdel-Shafy and Allam 2013; Hassan et al. 2017; Okely et al. 2021), which reported these species from imported camels in camel markets in Egypt. Phylogenetic analysis showed relatedness between the study sample sequences and the same species from other African countries such as Sudan, Guinea-Bissau, and Uganda. No *Amblyomma* species were collected from cattle because the cattle examined during this study were native to Egypt and not imported from other countries. As in the previous studies, *Amblyomma* species were not recorded from native cattle in Egypt. However, there are studies reporting *A. lepidum* and *A. variegatum* from imported cattle to Egypt (Robinson 1926; Hoogstraal 1956; Okely et al. 2022). Hence our results and previous studies suggest that native cattle were not infected by *Amblyomma* spp. in Egypt.

Illegal wildlife trade is a practice bringing several ecological and public health concerns, such as the spreading of zoonotic pathogens and their diseases continue to emerge and the introduction of exotic animals into new geographical areas (Brianti et al. 2010; Bezerra-Santos et al. 2021). There is growing concern that international trade without existing legislation and regulations will contribute to a loss in biological diversity and the extinction of several animal species worldwide by emerging novel infectious diseases (Smith et al. 2009). The trade in live reptiles has increased in recent years worldwide, which threatens wildlife conservation and public health (Franke and Telecky 2001). International trade in exotic reptiles increases the chance of ectoparasites and their pathogens to be introduced into new geographical locations (Burridge 2001). Tick species are the most reported parasites on internationally traded exotic reptiles (Mihalca 2015) and investigated infectious agents in imported reptiles and ectoparasitic ticks are a public health concern (Takano et al. 2010).

The number of ticks introduced to new exotic locations has increased and caused problems even in developed countries (Burridge 2001). The importation of exotic ticks into a new geographic area via reptiles has been reported in several countries worldwide. For example, In Italy, tortoises imported from North Africa were infested by ticks (Brianti et al. 2010); Mihalca (2015) reviewed the ticks imported to Europe with exotic reptiles, and the tick species belonged to the genera *Hyalomma* and *Amblyomma*. Several *Amblyomma* species have been reported in Poland for the first time by the international trade in reptiles (Nowak 2010). In the USA, *Amblyomma dissimile* was identified for the first time from imported reptiles (Pietzsch et al. 2006). Recently, there has been extensive trade of exotic reptiles, especially snakes as pets in Egypt (Satour and Dewir 2018; Jensen et al. 2019). Herein, the first record is reported for the *A. latum* from an African *Python* imported to Egypt.

Phylogenetic analysis showed that the collected *A. latum* sequence is closely related to specimens from South Africa, despite some population-level sequence divergence. Distinct branch length but the high similarity between Egyptian and South African *A. latum* (Fig. 3) suggest some minor population-level divergence effects over geographic distance, possibly due to the limited dispersal ability of reptile hosts. Historically, *A. latum* was detected outside the Afrotropical zoogeographic region and introduced to different parts of the world probably due to the international trade in live reptiles (Guglielmone et al. 2014). *Amblyomma latum* was collected from imported pythons in Florida, USA (Nearctic region) (Burridge et al. 2006; Corn et al. 2014) and *Python regius* imported into Argentina and Chile (Neotropical region) (González-Acuña et al. 2005). In Japan (Palaearctic region), this species infested *P. regius* imported from Ghana (Goka et al. 2013; Andoh et al. 2015) and *Phelsuma dubia* imported from Madagascar (Qiu et al. 2021). *Amblyomma latum* was collected from *Varanus exanthematicus* imported from Ghana to Poland (Palaearctic region) (Nowak 2010).

Finally, this study recommends the monitoring of tick species and their pathogens on imported wild and domestic animals from different ecological zones in Egypt.

## Electronic supplementary material

Below is the link to the electronic supplementary material.

Supplementary Material 1
