# Supplementary material for: Molecular screening of Amblyomma species (Acari: Ixodidae) imported from African countries to Egypt, with the first report of Amblyomma latum from the ball python, Python regius (Squamata: Pythonidae)
Source: Exp Appl Acarol. 2023 Aug 8;91(1):123–32. doi: 10.1007/s10493-023-00829-9 (PMC10462515; doi:10.1007/s10493-023-00829-9)
Supplement: Supplementary file 1 — Supplementary Material 1 [file 10493_2023_829_MOESM1_ESM.docx]

**Supplementary material**


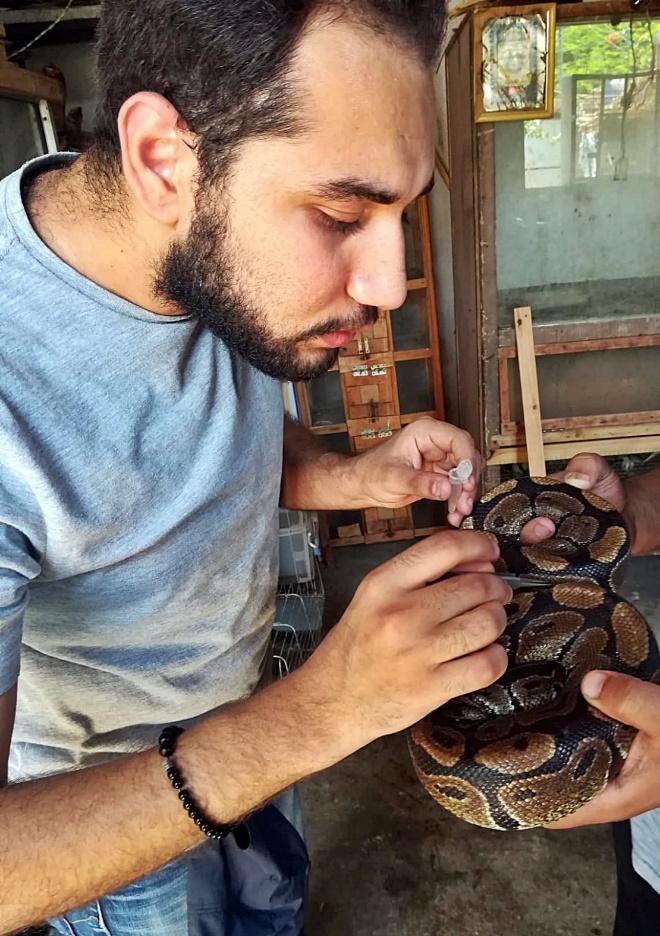
**Figure S1** Collecting tick from the ball python, *Python regius* from Faiyum, Egypt by the last author.
